# *Mycobacterium avium* subsp. *paratuberculosis* Virulence: A Review

**DOI:** 10.3390/microorganisms9122623

**Published:** 2021-12-19

**Authors:** Judah Ssekitoleko, Lonzy Ojok, Ahmed Abd El Wahed, Joseph Erume, Ahmad Amanzada, ElSagad Eltayeb, Kamal H. Eltom, Julius Boniface Okuni

**Affiliations:** 1 College of Veterinary Medicine, Animal Resources and Biosecurity, Makerere University, Kampala P. O. Box 7062, Uganda; jsekitoleko2810@gmail.com (J.S.); lonzyo@yahoo.com (L.O.); erujoseph@yahoo.com (J.E.); 2 Department of Livestock Health Research, Rwebitaba Zonal Agricultural Research and Development Institute, National Agricultural Research Organisation, Entebbe P. O. Box 295, Uganda; 3 Department of Pathology, Faculty of Medicine, Gulu University, Gulu P. O. Box 166, Uganda; 4 Institute of Animal Hygiene and Veterinary Public Health, Leipzig University, D-04103 Leipzig, Germany; 5 Department of Gastroenterology and Gastrointestinal Oncology, University Medical Centre Goettingen, D-37075 Goettingen, Germany; ahmad.amanzada@med.uni-goettingen.de; 6 Ibn Sina Specialised Hospital, Mohammed Najeeb St., Khartoum 11560, Sudan; sagadgady@yahoo.com; 7 Faculty of Medicine, Al Neelain University, 52nd St., Khartoum 11112, Sudan; 8 Unit of Animal Health and Safety of Animal Products, Institute for Studies and Promotion of Animal Exports, University of Khartoum, Shambat, Khartoum North 13314, Sudan; keltom@daad-alumni.de

**Keywords:** *Mycobacterium avium* subspecies *paratuberculosis*, Johne’s disease, virulence, pathogenesis

## Abstract

To propose a solution for control of *Mycobacterium avium* subsp*. paratuberculosis* (MAP) infections in animals as well as in humans, and develop effective prevention, diagnostic and treatment strategies, it is essential to understand the molecular mechanisms of MAP pathogenesis. In the present review, we discuss the mechanisms utilised by MAP to overcome the host defense system to achieve the virulence status. Putative MAP virulence genes are mentioned and their probable roles in view of other mycobacteria are discussed. This review provides information on MAP strain diversity, putative MAP virulence factors and highlights the knowledge gaps regarding MAP virulence mechanisms that may be important in control and prevention of paratuberculosis.

## 1. Introduction

*Mycobacterium avium* subsp. *paratuberculosis* (MAP) is a weakly Gram-positive acid-fast bacterium which causes Johne’s disease or paratuberculosis in animals especially ruminants [1,2]. It is also implicated in the cause of Crohn’s disease (inflammatory bowel disease) in humans [3]. Paratuberculosis is one of the most serious infectious diseases of dairy cattle worldwide, causing considerable loss in production in terms of reduced milk yield, reduced weight gain, culling/deaths and increased cost of disease control [4].

Among the attributes for a potentially pathogenic bacterium are its ability to gain access to a susceptible host, establish itself through evasion of the host defense mechanisms and multiply in the host, leading to disease or tissue damage and eventual dissemination to other hosts [5]. Genetic factors that enable the organism to achieve these pathogenic attributes are considered to be virulence factors. A lot still remains to be understood regarding the pathogenesis of MAP and its virulence in its numerous hosts. There are reports of differences in virulence among the various strains as well as differences in host susceptibilities [6]. This review is an attempt to elucidate the basis and mechanisms of differential virulence of MAP strains and isolates as reported to date.

## 2. Taxonomic Classification of MAP

*Mycobacterium avium* subsp. *paratuberculosis* is a Gram-positive bacillus, aerobic, nonmotile, non-spore-forming and acid-fast bacterium belonging to the genus *Mycobacterium* of the family Mycobacteriacea and is a member of the *Mycobacterium avium* complex (MAC) [7]. MAC are widely distributed in the environment, in healthy animals and humans and do not usually cause disease unless the host is debilitated or immunocompromised. Other members in the MAC complex include: *M. avium avium*, *M. avium silvaticum*, *M. colombiense* and *M. intracellulare*. They belong to the slow-growing group of mycobacteria, taking between 8–16 weeks to produce visible colonies on solid media such as Egg-based media such as Löwenstein–Jensen medium, Herrold’s egg-yolk medium and synthetic media such as Middlebrook 7H9, 7H10 and 7H11, though some strains may take up to 6 months [2,6]. MAP is classified biochemically as being dependent on externally provided mycobactin, though some strains have been found to be mycobactin independent.

## 3. MAP Strain Diversity

**Figure 1:**
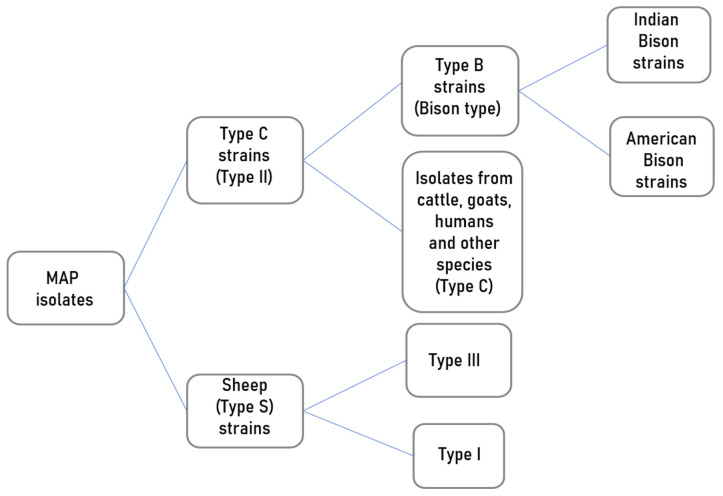
An illustration of MAP strain differentiation based on whole genome SNP-based phylogenetic analysis [13].

Principally, three broad MAP strain groups are recognised depending on their growth characteristics, colony pigmentation and host associations: Sheep type or “Type S”, the Cattle type or “Type C” and the Bison or “B-type” [8]. Some of the molecular techniques used in MAP strain typing for identification of genetic diversity include: IS900 restriction fragment length polymorphism (RFLP) [9], IS1311 RFLP, IS1311 polymerase chain reaction–restriction enzyme analysis (PCR-REA) [10], pulsed-field gel electrophoresis (PFGE) [11]; short sequence repeat (SSR) [12], mycobacterial interspersed repetitive unit-variable number tandem repeat (MIRU-VNTR) analysis [13] and single-nucleotide polymorphism (SNP) typing [14]. These typing techniques have divided MAP into Type I, II and III (Figure 1). Type I is predominantly isolated from sheep (Type S) and Type II is for isolates from cattle and other animals as well as humans. Type III is a subgroup of Type S. Based on SNP analysis of the IS1311, MAP first isolated from bison from Montana, USA, has been classified as Type B [15]. With further molecular analysis, this bison type was found to differ from a similar type from India giving rise to the Indian bison type [16]. Type B strains are a subtype of Type C but are not restricted to the bison. Type III and Type I appear to be subgroups of Sheep type [13]. Recent studies recommend that MAP characterisation should be based on whole genome sequence (WGS) analysis of genetic markers such as insertion elements, repetitive sequences and single nucleotide polymorphisms (SNP) [13,17] for better resolution, although this would be very expensive for small epidemiological studies.

## 4. MAP Pathogenesis

### 4.1. Transmission

MAP is transmitted in cattle mainly through the fecal–oral mode and infection occurs mostly in young calves [1,18]. Risk factors for MAP transmission include: importation or introduction of new animals whose MAP infection status is unknown, pooled milk feeding of calves and lack of or failure to use maternity pens on the farm [19,20]. Infected animals shed MAP in manure, colostrum and milk and young animals become infected through ingestion of colostrum and milk from the infected animals, contaminated water and pasture [21,22]. Upon ingestion, MAP enters the intestinal wall through the ileal mucosa via the microfold (M) cells residing in the Peyer’s patches [23]. It has also been demonstrated that MAP can cross the gut mucosa even in areas without Peyer’s patches entering through enterocytes [24]. Here, they resist intracellular degradation and eventually get phagocytosed by subepithelial macrophages. Infected macrophages migrate to local lymphatics and spread to regional lymph nodes where the organisms stimulate inflammatory and immunological responses [25,26].

### 4.2. Immune Responses

Infected macrophages stimulate T-helper lymphocytes and clonal expansion of two T-helper cell subpopulations: T helper 1 (Th1) and T helper 2 (Th2), which secrete different cytokines [25,27]. The Th1 cells produce pro-inflammatory cytokines such as interferon gamma (IFNγ), interleukin 2 (IL-2), IL-12 and tumour necrosis factor alpha (TNFα) while Th2 cells produce anti-inflammatory cytokines such as IL-4, IL-5, IL-6, IL-10 and IL-13 [28,29]. These cytokines are believed to orchestrate cell-mediated and humoral immune responses that aim to contain the intracellular infection but unfortunately end up augmenting the disease process. The early immune response to MAP infection consists of numerous infected macrophages with increased amounts of adhesion molecules, which result in formation of granulomas in which the bacilli remain secluded for a long period of time [27]. The animal in this state presents no clinical symptoms and it can remain in this subclinical phase for a period of 2–5 years while the bacilli are contained in macrophages and microscopic granulomas [30,31]. Infected animals may not manifest clinical disease throughout their lifetime, yet they continue to contaminate the environment through shedding of MAP [32,33,34]. What determines the length of this duration both at host and pathogen levels is still unknown. With the failure of macrophages to destroy MAP, the organisms begin to multiply intracellularly with production of immunoglobulin G1 (IgG1) antibodies characteristic of the late humoral response in MAP infection [28].

## 5. Fate of MAP inside Macrophages

Mononuclear phagocytes, principally macrophages, serve as the intracellular niche for MAP survival and multiplication [35]. Macrophages possess several receptors involved in mycobacterial uptake which include complement receptors (CR1, CR3 and CR4), mannose receptors, immunoglobulin receptors and the scavenger receptors [36,37,38]. Reports suggest that complement opsonisation is important for the uptake of MAP by bovine mononuclear phagocytes and that these phagocytes can synthesise and secrete complement proteins to opsonise particles for phagocytosis [39].

The innate immune system also employs germline-encoded pattern-recognition receptors for the initial detection of microbes. There are several classes, some of which include the Toll-like receptors (TLRs) and Nucleotide-binding oligomerisation domain-like receptors (NLRs) [40]. Antigen-presenting cells such as macrophages express these TLRs, which initiate immune responses mediated by different cytokines. Reports have indicated that TLRs and NLRs are involved in the recognition of MAP by the innate immune system [41].

**Figure 2:**
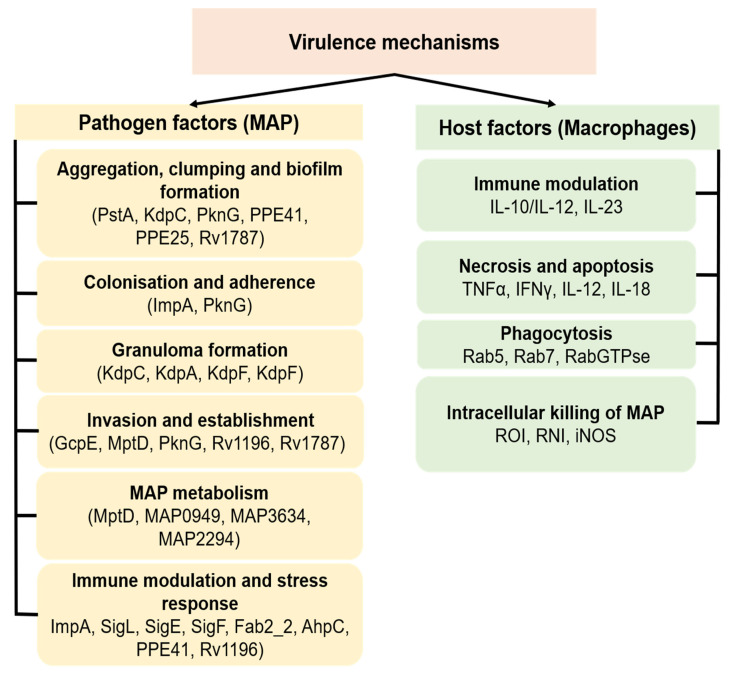
Schematic representation of putative MAP virulence mechanisms and associated host factors. The virulence mechanisms enable MAP to gain entry, survive, multiply and cause disease in the host by interacting and sometimes disabling the host defense mechanism and metabolism. Because limited information is available concerning the expression and actions of virulence factors in MAP, most of the current understanding of MAP virulence is imputed from the functions of such factors in related mycobacteria.

Mechanisms of virulence responsible for MAP colonisation, entry and persistence in macrophages and eventual disease development remain elusive. Several mycobacterial factors are believed to interplay and interact with host factors to determine the virulence and pathogenesis of MAP within the animal host (Figure 2). Once inside macrophages, MAP survives and proliferates in the phagosome using means which are not fully understood. One such means is the ability of MAP to disable the reactive oxygen anion intermediates (ROI) such as superoxide anions, hydrogen peroxide and hydroxyl radicals [42], which have a mycobactericidal effect. MAP secretes superoxide dismutase, which is a possible counter mechanism for protection of the bacilli in macrophages [43]. Macrophages also produce reactive nitrogen intermediates such as nitric oxide (NO) when stimulated with IFN-γ and TNF-α which are also known to have a mycobactericidal effect [44]. Macrophages infected with *M. tuberculosis* (MTB) have been shown to inhibit recruitment of inducible nitric oxide synthase (iNOS) in phagosomes containing mycobacterium as a measure to counteract NO production [45]. A similar mechanism is believed to work in macrophages infected with MAP, though this requires further investigation since [46] found little evidence of iNOS activity in Johne’s disease lesions.

Another microbicidal mechanism by phagocytes is the phagosome–lysosome fusion to form a functional phagolysosome in which the lysosomal vacuoles containing hydrolytic enzymes kill and degrade invading microbes [47,48]. Live MAP has been shown to perforate this vacuole by secreting lipids that create pores in the membrane surrounding the vacuole. Mycobacteria are also known to interfere with phagosomal maturation through inhibition of the ATP-dependent proton pump (H+-ATPase) which maintains an acidic environment necessary for optimal activity of the hydrolytic enzymes [49]. MAP cells have been shown to inhibit phagosome–lysosome fusion and phagosomal acidification in murine cells [50,51]. This is through blocking of Rab5 activity, therefore preventing the fusion of early endosomes. Activation of MAP-infected murine macrophages with IFN-γ and lipopolysaccharide (LPS) resulted in enhanced phagosome–lysosome fusion and increased killing of MAP cells intracellularly as compared to the unstimulated [52]. MAP cells have been shown to stimulate production of IL-10, an anti-inflammatory cytokine, which counteracts the effects of IFN-γ in macrophage infection and aids MAP survival [53]. Consequently, downregulation of IL-10 gene resulted in reduced survival of MAP but also increased production of INF-γ in bovine peripheral blood mononuclear cells (PBMC) [53,54]. Similar reports have showed that infection of bovine macrophages with MAP resulted in reduced apoptosis of macrophages [51,55] which enhances MAP survival.

Mycobacteria, unlike other pathogenic bacteria, lack the classical virulence factors such as toxins; they uses other virulence mechanisms which enable them to survive in the macrophage intracellular environment [56]. The mechanisms utilised by MAP to enter and survive in the host macrophages include resistance to intracellular degradation by macrophages and inhibition of apoptosis and interference with cytokines production by macrophages. Cytokines control different processes in the cell through protein regulation to overcome the infection. MAP, however, modifies the response of macrophages to infection so that it is able to survive inside the macrophage [26].

Although the mechanisms for the intracellular survival of MAP within macrophages are not entirely understood, several reports suggest that these mechanisms have a genetic basis [8,57]. Khalifeh and Stabel reported that formation of persistently infected macrophages is associated with upregulation of IL-10, transforming growth factor beta (TGFβ) and reduction in IFNγ [58]. It has been shown that MAP interferes with CD40 signalling, which is mediated by IL–12P40 and inducible Nitric oxide synthase (iNOS) in monocytes-derived macrophages [59]. The CD40 pathway is responsible for the activation of macrophages by the T cells. Failure of T cell activation and consequently macrophage activation leads to persistently infected macrophages incapable of destroying the pathogen that instead act as a vehicle for propagation and dispersal of the organism leading to infection [25,60]. Activated macrophages are capable of killing MAP and control their growth; however, at high multiplicity of infection, MAP is cytotoxic to macrophages and also inhibits apoptosis [61,62].

## 6. MAP Virulence Factors

Virulence factors facilitate adhesion, invasion and colonisation of host cells by pathogenic bacteria; they include enzymes of several lipid pathways, cell surface protein regulators and signal transduction systems molecules (Figure 2). Bacterial virulence genes may be located on transmissible genetic elements such as transposons, bacteriophages, plasmids or within the bacterial genome organised in a contiguous region known as the pathogenicity island [63]. A gene will probably contribute to virulence if it is present in a pathogen but absent in a closely related nonpathogenic organism or inactivation of the gene results in attenuation of the virulence phenotype and replacement with an intact copy of the inactivated gene restores virulence [64].

### 6.1. Factors That Facilitate Adhesion, Colonisation, Entry and Persistence

From studies so far conducted, it has been demonstrated that certain genetic elements within the pathogen genome are responsible for virulence. Once removed or disrupted, the organism displays an attenuation phenotype. The disruption of genes can be achieved experimentally by means of insertion or deletion mutagenesis and construction of transposon mutant libraries. Using a transposable element Tn5367 introduced into the MAP genome by means of a conditionally replicating mycobacteriophage phAE94, Harris and colleagues generated a library of 5620 MAP insertion mutants and also demonstrated that the transposon insertion sites are distributed relatively randomly throughout the MAP genome [65]. In a related study, Shin and coworkers constructed a library of transposon mutants whose genes were phenotypically involved in membrane transport protein, iron, tryptophan, or mycolic acid metabolic pathways [66]. In the aforementioned study, the transposable element Tn5367 was used to generate a library of MAP mutants. A total of 5060 mutants were generated, of which 1150 mutants were characterised by sequencing and bioinformatics analysis. Out of these, using a mouse model of paratuberculosis, seven novel virulence determinants were identified. Bacteriological and histological analyses employed in the study were able to categorise the putative colonisation virulence factors into two classes: those that exhibited impaired organ colonisation and low inflammatory scores; and those that showed low colonisation rates but at a later stage of infection, implying a role for persistence in macrophages. These genetic factors are involved in *M. tuberculosis* (MTB) virulence and might have significance in MAP virulence as well.

In Table 1, we present a list of virulence factors of MAP and related mycobacteria which are believed to play a role in MAP virulence and pathogenesis. The gene *gcpE* encodes a protein involved in isoprenoid biosynthesis via the mevalonate-independent 2-C-methyl-D-erythritol-4-phosphate (MEP) pathway [67]. It is considered an essential gene in *Escherichia coli (E. coli)* [68]. In a study using a calf model in which MAP strains were deposited in the ileum, mutants of the *gcpE* gene were unable to traverse the intestinal barrier to the mesenteric lymph nodes, unlike the wild-type strain. This indicated that the mutant was less invasive, throwing more light on the process of MAP pathogenesis and virulence mechanism [69]. However, more information is required on the probable use of the *gcpE* gene as a potential target for vaccine development in paratuberculosis control.

The nonribosomal peptide synthetase gene (*pstA*) encodes an enzyme involved in glycopeptidolipid biosynthesis which is associated with biofilm formation [70]. It has been described in mycobacteria such as MTB, *M. smegmatis* and *M. avium* [71,72,73]. Glycopeptidolipids are species-specific mycobacterial lipids and have been demonstrated to be major constituents of cell envelopes of several nontuberculous mycobacteria, both pathogenic and nonpathogenic [74]. Biofilm formation is believed to contribute to bacterial virulence by inducing a persistent source of infection and may contribute to antibiotic resistance [75]. According to Shin and coworkers, *pstA* mutants showed significant reduction in tissue colonisation in a mouse model of paratuberculosis [66]. In a more recent study, *pstA* mutants were shown to be deficient in the ability to form biofilm when compared with the intact MAP strain [70], an indication that PstA could contribute to biofilm formation in MAP. In the same study, it was observed that *pstA* mutants also exhibited significant reduction in bacilli length compared to the parent strain, which raises the question of whether cell elongation could contribute to biofilm formation. The exact role of PstA in MAP pathogenesis requires further scrutiny.

The *kdpC* gene is a probable potassium-transporting ATPase C chain which encodes an inducible high-affinity potassium uptake system of *E. coli.* It is part of the Kdp complex, which are membrane bound subunits comprising the KdpC, KdpF, KdpB and KdpA [76]. The role of the *kdpC* gene in mycobacterial pathogenesis is not yet clear. From the study by [66], mutants for this gene were deficient in the ability to colonise mouse organs (liver and intestines) and this could possibly be due to a defect in the potassium-shuttling mechanism which resulted in the attenuation phenotype exhibited by the mutants. The *kdpC*-deficient mutants showed limited granuloma formation compared to the wild types.

Similarly, disruption of the *papA2* gene in MAP resulted into attenuation in a murine model of paratuberculosis [66]. The *papA2* gene is a member of the conserved polyketide synthase-associated protein (Pap) family which encodes virulence-enhancing lipids of MTB [77].

The *impA* gene encodes an inositol monophosphatase protein which earlier studies showed that it played a role in cell wall permeability in *M. smegmatis* and in the synthesis of phosphatidylinositol dimannoside [78,87]. On the other hand, *fabG2_2* gene encodes a putative oxidoreductase activity in MAP [79]. Both mutants of *impA* and *fabG2_2* genes exhibited low colonisation levels late in the infection experiment, which implied that they could possibly play a role in bacterial defense in the persistence stage of MAP infection [66].

The *umaA1* gene, which encodes a mycolic acid methyltransferase, is involved in cell wall biosynthesis [79]. Disruption of *umaA1* resulted in reduced colonisation levels of mutants in mice [66]. This is, however, in contrast to a similar model for MTB in which *umaA1* mutants exhibited a hypervirulence phenotype [88] and this may suggest a difference in role played by the gene in MTB from that of MAP. MAP *umaA1* together with *fabG2_2* gene mutants have been tested for their vaccine potential in mice and were found to induce a MAP specific IFN-γ important for eliciting cell-mediated immunity. The *umaA1* mutants further induced production of IL-17a, a cytokine important for mycobacterial protective immunity. Indeed, mice vaccinated with the *umaA1* and *fabG2_2* gene mutants exhibited significant reduction in organ colonisation and low histological scores compared to control animals when challenged with a virulent MAP strain [80].

Though the above-described virulence genes have been identified, there is little understanding of the pathways through which they act and whether the same effects observed in vitro and in mouse models can be observed in ruminant models of paratuberculosis. Moreover, similar genes in closely related species may play different roles in pathogenesis due to differences in gene organisation on the genomic island, as was observed in MAP and *Mycobacterium avium* subsp*. avium* [89,90]. How virulence factors affect host–pathogen interactions is still only left to speculation. These probable virulence determinants could represent novel functional classes necessary for mycobacterial survival during infection and could provide suitable targets for vaccine and drug development.

### 6.2. Factors Which Affect Metabolism of MAP

Different pathogenic bacteria have been demonstrated to undergo metabolic adaptations that enable them to utilise the host metabolites that would otherwise have been toxic. An example is *M. tuberculosis,* which has the ability to break down the host fatty acids, detoxify the products and utilise the resulting molecules to acquire carbon during intracellular infection. The ability of pathogens to acquire nutrients within the host environment is part of the crucial processes contributing to bacterial virulence [91].

MAP has been shown to undergo significant genetic modifications during host–pathogen interaction within macrophages. One such modification is iron limitation, which triggers nitric oxide build-up linked to nitric oxide synthase production, which prompts MAP to enter into an iron sequestration program. This kind of pathway is likely to contribute to MAP virulence by aiding MAP establishment and long-term survival within host macrophages [92]. However, it is not known how MAP influences the host metabolic state for its benefit and whether the genetic factors contributing to MAP virulence are also involved in these processes. To date, only a couple of genes have been shown to modulate MAP virulence within macrophages (Figure 2).

Meissner and others [81] studied the role of MAP *mptD* gene (*map3733c*) to deduce its potential role in the host during MAP infection. The *mptD* gene whose operon is predicted to encode a putative ATP-binding cassette transporter belongs to a small group of functionally uncharacterised genes. Macrophage infection experiments were performed in which the gene was observed to have a significant role in MAP adaptation during early infection. Metabolic profiling revealed profound disorders in lipid metabolism, hence pointing to the probable importance of *mptD* gene in metabolic adaptation required for MAP persistence in the host.

In a recent study, Philips and others used an *Acanthamoeba castellanii* (amoeba) model to predict virulence mechanisms of MAP [86]. A library of gene knockout mutants generated by Mycomart7 (Mmt7) transposon was used to identify MAP clones that can either enhance or inhibit the amoeba metabolic activity, and this mirrored the pattern of MAP survival or attenuation in macrophages. They observed that MAP mutants that induced high amoeba metabolic activity were defective in the intracellular growth inside macrophages. From bioinformatic analysis of mutant sequences, several genes were identified that could possibly be responsible for the altered ability for survival of MAP mutants in the RAW 264.7 macrophages [86].

The *map3893c* gene encodes the serine/threonine-protein kinase G (PknG) which is a well-characterised virulence factor in MTB where it is known to contribute to biofilm development and granuloma formation [82,93]. It is also known to block the recruitment of active Rab711-GTP to phagosomes containing the pathogen, thereby inhibiting the phago-lysosome fusion [94,95]. It appears that the role of PknG in MAP pathogenesis contributes to macrophage phosphorylation signalling and other adaptor molecules by inducing an immune response through production of IFN-γ [83]. The exact role of PknG in MAP is not fully understood, but because it shares high homology with that of the MTB complex, it is anticipated to perform similar roles. This, however, needs further investigation.

The second macrophage-related putative virulence factor is MAP0949, a probable diguanylate cyclase whose absence results in severe attenuation of MAP during macrophage infection. Its wild phenotype is, however, restored once the disrupted gene is complemented by the functional gene [86]. In other bacteria, it has been postulated that it is involved in stimulating degradation of second messenger cyclic di-GMP (c-di-GMP) involved in bacterial cell surface adhesions [84]. The c-di-GMP and c-AMP second messenger signalling molecules control expression of a variety of environmental and quorum sensing signals as well as regulating several key virulence mechanisms required for bacterial adaptation and evasion of the host immune system [85,96,97].

Inducible nitroxide synthase (iNOS) is an important enzyme responsible for the activation of reactive nitrogen oxide species that kill intracellular mycobacteria. Virulence of mycobacteria is probably mediated by the *map2291* gene, a haemoglobin-like oxygen carrier (*glbO*) which encodes the globin protein occurring across the *Mycobacterium avium* complex (MAC). It is 86% homologous to MTB oxygen-binding *glbO* which have a function related to oxygen affinity and reactivity [98]. MTB-truncated haemoglobin O (trHbO) displays moderate NO-scavenging activity which signifies involvement in both NO detoxification and aerobic respiration [99]. Since *map2291* consists of MTB *glbO*-like domain, it can be hypothesised that the *map2291* gene prevents intracellular killing of MAP by protecting the pathogen from microaerophilic conditions and oxidative stress [86]. Moreover, one study also showed evidence of limited iNOS activity in MAP granulomas [46].

Virulence of MAP may also be determined by the *map3634* gene, which encodes a hypothetical protein that contains IgD-like repeat domain of mycobacterial L,D-transpeptidases and is responsible for the final polymerisation steps involved in the formation of glycan strands and cross-linking peptide stems of the peptidoglycan cell wall in most bacteria [100]. Inactivation of these transpeptidases has been demonstrated to be detrimental to MTB and *M. abscessus* [101,102], signifying its importance as a virulence factor in these organisms. MAP mutants lacking *map3634* gene exhibit reduced survival rate in macrophages, presumably due to defective bacterial cell wall synthesis [86].

### 6.3. Factors That Affect Aggregation and Clumping

The PE/PPE proteins are among the probable factors contributing to MAP virulence. The PE/PPE proteins are large families of proteins with each member sharing a conserved N-terminal domain with the characteristic motifs Proline–Glutamate (Pro–Glu or PE) or Proline–Proline–glutamate (PPE) that are an important domain in mycobacteria and have been for long believed to play a role in virulence [103]. They have been well characterised in MTB, though they also occur in other mycobacteria such as *M. leprae*, *M. avium* and *M. bovis* [104]. They are thought to be virulence factors responsible for evasion of host immune responses and a potential source of antigenic variation in MTB [105]. It is reported that some PE/PPE families are deleted from avirulent *Mycobacterium tuberculosis* [106]. Zheng and colleagues also showed that a number of PE/PPE genes found in virulent strains of MTB contain single nucleotide variations [107]. Differential expression of PE/PPE genes in *M. tuberculosis* and *M. bovis* seems to suggest host specificity. At least one *M. tuberculosis* PE protein is known to cause cell death while another causes apoptosis of host macrophages [108]. There is some evidence that inactivation of the PE-polymorphic GC-rich repetitive sequence (PE-PGRS) gene in *M. bovis* BCG strain will lead to loss of cell aggregation (clumping), dispersed growth and reduced infection of macrophages [109]. The iron-dependent regulator (IdeR) of MTB has been reported to control several PE/PPE proteins such as Rv0279, Rv0285 and Rv2123, and the fact that iron is necessary for virulence points to the PE/PPE proteins being virulence factors [110,111]. In MAP the PE/PPE family comprises 1% of the genome and lacks the intact PE-PGRS subfamily present in MTB and other mycobacteria such as *M. bovis* and *M. marinam* [79].

The exact role of PE/PPE proteins in MAP virulence has, however, not yet been elucidated. PE-PGRS family proteins Rv0834c, Rv3097c, Rv097 are expressed within 24 h post infection in macrophages and are believed to aid the establishment of the infection in macrophages [105]. It is suggested that PE-PGRS Rv1759 binds to the cytoskeletal protein, fibronectin thereby facilitating entry of MAP into the host through opsonisation [112]. Another PE-PGRS protein, Rv1787 (PPE25) has been shown to be important for growth of *M. smegmatis* within macrophages, while Rv1196 (PPE18) and PPE44 trigger responses that favour a switch from Th1 to Th2 by shifting IL-10/IL-12 balance and down regulating IL-12/TNFα. Because of the high degree of polymorphisms among the PE-PGRS genes observed even among the different strains of mycobacteria [113], they are potential determinants of differential virulence in mycobacterial strains. As has been recommended for other mycobacteria, the macrophage infection model of MAP can be used to answer questions about activation of various PE/PPE genes, their regulators and mRNA stability [111]. Another aspect of MAP pathogenesis and virulence is to understand how this organism modulates IL-10/IL-12 response.

### 6.4. Global Gene Regulators and Stress Induced Genes

Global gene regulators (GGR) are involved in the control of several other genes such as the sigma factors which have been shown to be important in regulating MAP virulence. In one of the studies by [92], of the 19 alternative sigma factors encoded in MAP [79], only SigL was induced at an early stage when subjected to stressors to mimic the host microenvironment such as oxidative and cell wall stressors. It has been suggested that *sigL* regulates the synthesis of cell envelope lipids and is responsible for modification of secreted proteins in MTB [114]. The importance of *SigL* in MAP survival in macrophages during early adaptation and the ability of mutants to produce protective immune response against paratuberculosis [57] further highlighted the importance of GGR in possible development of effective live attenuated vaccines.

Wu and colleagues studied the gene expression profiles of MAP under different stress conditions such as heat shock, acidity and oxidative stress. Several sigma factors such as *SigH*, *SigE* were differentially coregulated with a large number of genes. Deletion mutagenesis revealed attenuation of gene mutants that included; *lipN, lpqP, aceAB,* and *prrA* in a murine model of paratuberculosis which could participate in tissue colonisation indicating their role in MAP pathogenesis [115]. Pribylova and coworkers also reported that SigE, SigL, SigF, alkyl hydroperoxide reductase (AhpC) and the major membrane protein (MMP) are over expressed during heat stress in MAP [116]. Further analysis of the role of stress-induced genes in MAP pathogenesis and virulence is needed.

### 6.5. The Role of Polymorphism in the MAP Genome on its Virulence

Microbial genetic variations may be as a result of spontaneous mutations due to the unstable nature of the purine and pyrimidine bases, as a result of errors that may occur during replication or be induced by exposure to certain environmental factors such as UV light [8].

Whole genome sequence analysis of the MAP genome has revealed occurrence of large sequence polymorphisms (LSPs) emanating from deletions, insertions, inversions, duplications or dislocations. These events can cause DNA rearrangements resulting in major phenotypic changes such as acquisition of virulence genes through insertions. Other sources of genetic variations include: insertion sequences, repeat sequences and single-nucleotide polymorphisms (SNPs) [8]. The MAP genome comprises 19 different insertion sequences which are useful in mycobacterial species and subspecies differentiation such as IS1311 and IS900, which is MAP-specific [117]. Repeat sequences are also important in genomic typing for strain differentiation and molecular diagnosis such as the mycobacterial interspersed repetitive units–variable-number tandem repeats (MIRU-VNTRs) and short-sequence repeats (SSRs) [118]. SNPs are the substitutions of a single nucleotide with another or a deletion or insertion of one nucleotide. Single-nucleotide substitutions in protein synthesis can be synonymous—resulting in no amino acid change—or nonsynonymous, which results in amino acid changes. SNPs can be utilised in intra-strain differentiation and offer the greatest microbial genomic variation and may contribute to variations in pathogenicity among MAP strains [8,14,117]. Genetic variations, including single nucleotide polymorphisms in PE/PPE-PGRS genes, have been found to occur more frequently in virulent strains of *M. tuberculosis* compared to the avirulent ones. All these different polymorphisms could play important roles in differential virulence in MAP but further study is required to determine if this is true.

## 7. Conclusions

Differences in MAP strain virulence have been reported by several studies, but the slow growth of MAP and its long incubation period have made it very difficult to understand the mechanisms for its pathogenesis and virulence in its natural hosts. Most of the current understanding is based on inference from other mycobacteria, with a few studies attempting to replicate these studies using MAP itself. The key highlights of MAP virulence focus on genes such as the PE/PPE—PGRS family, mycobacterial protein kinases (*PknG*) and the modulation of IL-12/IL-10 switch during infection. There are still many knowledge gaps in our understanding of how differential expression, stability and secretion of these gene products affect MAP uptake, persistence, frustration of phagocytosis and toxicity, which are the hallmarks of mycobacterial virulence in its hosts. Future studies need to focus on elucidating the different protein pathways involved during mycobacterial pathogenesis.

## Figures and Tables

**Table 1:** Factors believed to influence virulence in MAP and other mycobacteria.

Virulence Factor	Name/Function	Probable Role in MAP Virulence	References
GcpE	Protein involved in isoprenoid biosynthesis	Involved in the MEP pathway. Important in tissue invasion during early MAP infection	[69]
PstA	Non-ribosomal peptide synthetase	Glycopeptidolipid biosynthesis and associated with biofilm formation	[70]
KdpC	Probable potassium-transporting ATPase C chain	An inducible high-affinity potassium uptake system. In MAP it has been associated with organ colonisation and granuloma formation	[66,76]
PapA2	Conserved polyketide synthase-associated protein	Virulence-enhancing lipids of MTB. In MAP it is associated with tissue colonisation	[66,77]
ImpA	Inositol monophosphatase protein	Involved in cell wall permeability and persistence in macrophages	[66,78]
FabG2_2	Putative oxidoreductase	Involved in colonisation and persistence in macrophages during MAP infection	[66,79]
UmaA1	Mycolic acid methyltransferase	Involved in cell wall biosynthesis and tissue/organ colonisation	[79,80]
MptD (MAP3733c)	Putative ATP binding cassette transporter	Important in MAP adaptation during early infection through lipid metabolism	[81]
PknG (MAP3893c)	Serine/threonine protein kinase G	Contributes to biofilm and granuloma formation in MTB. In MAP it induces production of IFNγ leading to macrophage phosphorylation	[82,83]
MAP0949	Probable diguanylate cyclase	Involved in bacterial cell surface adhesions. Important in adaptation and evasion of the host immune system.	[84,85]
MAP2291	Haemoglobin-like oxygen carrier—glbO	Protects MAP against oxidative stress	[86]
MAP3634	Hypothetical protein of mycobacterial L,D-transpeptidases	Involved in bacterial cell wall synthesis through polymerisation of peptidoglycans	[86]

## Data Availability

All data are available in the manuscript text.
